# Decisive Factors for a Greater Performance in the Change of Direction and Its Angulation in Male Basketball Players

**DOI:** 10.3390/ijerph17186598

**Published:** 2020-09-10

**Authors:** Francisco J. Barrera-Domínguez, Bartolomé J. Almagro, Inmaculada Tornero-Quiñones, Jesús Sáez-Padilla, Ángela Sierra-Robles, Jorge Molina-López

**Affiliations:** 1 Faculty of Education, Psychology and Sport Sciences, University of Huelva, Avda. Tres de Marzo, s/n, 21071 Huelva, Spain; fbarreradominguez@gmail.com (F.J.B.-D.); almagro@dempc.uhu.es (B.J.A.); inmaculada.tornero@dempc.uhu.es (I.T.-Q.); jesus.saez@dempc.uhu.es (J.S.-P.); sierras@dempc.uhu.es (Á.S.-R.); 2 Institute of Nutrition and Food Technology, Biomedical Research Centre, Health Sciences Technological Park, University of Granada, 18010 Granada, Spain

**Keywords:** team sports, category, force–velocity profile, reactive strength index, body composition, functional movement

## Abstract

A study was made to initially evaluate whether the age category directly could influence anthropometric measurements, functional movement tests, linear sprint (30 m) and strength. Moreover, and as the main purpose, this study aimed to examine the relationship between the time execution and angles in different changes of direction (COD) test with the analyzed sport performance variables. A total sample of 23 basketball players (age: 17.5 ± 2.42 years; height: 184.6 ± 6.68 cm; body weight: 78.09 ± 11.9 kg). Between-groups’ comparison explored the differences between basketball categories (Junior, *n* = 12; Senior, *n* = 11). The COD variables were divided by the time execution into low responders (LR) and high responders (HR) to establish comparisons between groups related to COD time execution. Pearson’s correlation coefficient was used to establish correlations between different CODs and sport performance variables. The results showed a greater influence of age category upon COD performance, especially when the cutting angle was sharper (7.05% [Confidence limits (CL) 90%: 2.33; 11.99]; Quantitative chances (QC) 0/2/98), in which athletes need greater application of strength. Moreover, the sharper the angle or the larger the number of cuts made, the greater the relationship with the vertical force–velocity profile (−42.39 [CL 90%: −57.37; −22.16]; QC 100/0/0%). Thus, the usefulness of the f–v profile to implement training programs that optimize the f–v imbalance and the improvement of the COD performance in basketball players is suggested.

## 1. Introduction

Basketball is characterized by high-intensity actions in the form of jumps, accelerations, decelerations, and rapid changes of direction (COD) that are regarded as key components of this sport [1]. Basketball players have been found to make an average of 1050 movements during a competitive game, resulting in a change in speed or direction every two seconds, and demonstrating the highly intermittent character of basketball [2]. Also, elite athletes tend to cover a distance of 991 m with high-intensity actions, averaging 40–60 maximum jumps and 50–60 speed changes and CODs [2]. Specifically, 52% of all sprints during a basketball match involve fast CODs [3]; the efficiency of both ball and nonball CODs is, therefore, a critical component in sports performance.

Agility has been defined as a rapid whole-body movement with a change of speed or direction in response to a stimulus [4]. Agility comprises a process of perception and decision making that results in COD or speed, but a COD and speed that does not involve the perception of a stimulus and decision making is no longer agility but a COD or speed skill [5,6]. It has been argued that this COD skill is a fundamental prerequisite for success in modern sports [7]. Our main interest focused on COD as a component of agility. Many factors can influence the speed of COD due to its multifactorial character [6]. Among them, technical, anthropometric, and physical factors stand out [4], with both linear velocity and muscular activity of the lower limbs through strength and power being of particular relevance [8]. Based on previous evidence, all of these factors might vary between basketball players of different age categories or playing levels [9,10]. Although many studies have focused on COD and its relation to physical performance, to our knowledge no studies have examined all these variables simultaneously in one, same sample, which would allow analysis of the real influence of each parameter upon COD. To date, the literature offers only limited and conflicting results regarding the possible relationship between these physical parameters.

In this context, previous evidence examined isolated sports performance variables such as linear velocity finding an association with COD involving small angles [11]. Previous studies added that shorter sprints prior to COD did not allow high accelerations [12]. Furthermore, several authors [12,13] showed that in young basketball players shuttle sprinting with a COD of 180° both oxygen uptake and blood lactate concentration increases with the shuttle distance, while the metabolic cost decreases. Therefore, within the COD itself, the distance of sprinting carried out prior to the COD affects metabolic involvement, acceleration, and braking forces. Strength is another essential skill for optimal performance in COD actions [14]. Specifically, the countermovement jump (CMJ), together with the drop jump (DJ), is the most used test for evaluating strength in relation to COD [15]. However, there is no clear consensus about the results of this relationship between jumps and COD [16,17]. Moreover, previous studies have analyzed strength by separating the different phases of muscle contraction [18,19] as a strength. The present study considered analysis of the force–velocity (f-v) profile through different CMJs, where the stretch-shortening cycle was used (as in the COD), with loads from body weight (BW) itself up to 60 kg extra. In this way, we can analyze a broader spectrum of the force–velocity curve, with force being understood as a single physical capacity without differentiating between its different manifestations. To asses COD, specifically in basketball, the T-test is the most used [18,19,20,21], where the player has to cover a circuit in the form of a T with the aim of moving in all directions in the least time possible, moving forward, sideways, and backward (a similar situation to that encountered in a real game) [21]. To our knowledge, many studies carried out the 505, test with 180° COD [14,19] but other angulations have not been studied, this would be important because basketball is a sport that involves many COD with different angulations.

Therefore, the present study initially sought to evaluate whether the age category could influence anthropometric measurements, functional movement tests, linear sprint (30 m), and strength. Moreover, and as the main purpose, the present study aimed to examine the relationship between the time execution in different COD tests and angles with the analyzed sport performance variables. Such knowledge could be crucial for strength and conditioning practitioners.

## 2. Materials and Methods

### 2.1. Participants

A cross-sectional study was performed with 23 amateur basketball players aged between 16 and 27 years. The sample comprised two age categories: A junior group (*n* = 12) (age: 16.25 ± 0.45 years; height: 181.9 ± 6.36 cm; body weight: 72.02 ± 8.46 kg) and a senior group playing in the Spanish National Division (*n* = 11) (age: 19.00 ± 2.90 years; height: 187.6 ± 6.59 cm; body weight: 84.73 ± 11.87 kg). Subjects were eligible for inclusion if they had at least five years of previous experience, played at a federated level, no injuries during and at least six months before the study, and had regular training between three or four days per week during the competitive period. All players were trained by the same coach and strength-conditioning trainer, were in good health, and passed a medical examination before starting their basketball season. Data collection was conducted after the preseason, coinciding with the start of the competitive period. During and prior to the data collection period, the training duration of the athletes consisted of 7.1 ± 1.2 h/week of a basketball training regimen including both indoor and integrated conditioning exercises, in addition to competition in matches on weekends. The predesigned weekly training schedule of the basketball teams comprised the following: Day 1–strength training and individual technique; Day 2–integrated strength training and small side games; Day 3–offensive and defensive technical–tactical training; Day 4–match. All players were informed about the objectives of the study and gave consent to participation. The procedures followed the rules established by the Declaration of Helsinki. The present research was approved by the Andalusian Ethics Committee of Biomedical Research (reference number: FBD_UHU2020).

### 2.2. Procedure

During the preseason, familiarization sessions were held so that the players would have performed each test at least 3–6 times. During the measuring days, all tests were performed on the surface where they train and compete. All players were instructed to attend with good hydration and rest, without vigorous training in the previous 24 h, no caffeine, drink, or food intake in the three hours before the test, no alcohol intake in the previous 24 h, and urination immediately before the anthropometric measurement.

Prior to data collection, all subjects performed a standardized warm-up including a routine of 5 min of low intensity with ball, 5 min of hip mobility and core activation, 5 min of low-impact landings, accelerations, and plyometrics and, finally, 5 min of approximation to the tests, increasing the intensity in each repetition. The proposed tests were grouped together to be performed in four different sessions: (1) anthropometric measurements and weight-bearing dorsiflexion test (WB-DF), star execution balance test (SEBT), and Y balance test (YBT); (2) linear speed test using a 30-m sprint test (30S) and triple hop test for distance unilateral (THTU); (3) vertical force–velocity profile of each player using four CMJ at different loads and drop jump unilateral test (DJU) at a height of 30 cm; and (4) proposed COD test: Modified T-test and the 5 + 5 test with different angles at 45°, 90°, and 180°.

### 2.3. Anthropometric Measurements

Body height was measured with a stadiometer (0.1 cm, Secca 220, BLINDED). While this was done, the players remained static in the anatomical position with their heels together. The highest point of the head was taken as reference. Body composition measurements were taken by multi-frequency bioelectrical impedance (Inbody 230 Multi-frequency Segmental Body Composition Analyzer, Barcelona, Spain). The analyzer complied with the applicable European standards (93/42 European Economic Community (EEC), 90/384 EEC) for use in the medical industry. The impedance between the two feet was measured while an alternating current (50 kHz, 90 milliamps) was passed through the lower body. Participants were informed in advance of the conditions that had to be sustained prior to measurement: No alcohol for at least 24 h before the measurement, no vigorous exercise for at least 12 h before, no food or drink for at least three hours before, and urination immediately before the measurement. The following measurements were taken: Weight, body mass index (BMI), lean mass, and fat mass expressed as a percentage of total body fat.

### 2.4. Weight-Bearing Dorsiflexion Test (WB-DF)

Ankle dorsiflexion was evaluated through the Dorsiflex iPhone App (Apple Inc., Cupertino, CA, USA) [22]. Initially, each player stood with his hands on his hips, placing the leg to be measured forward and the opposite leg resting one foot behind. The test was performed without any type of footwear. While maintaining the position, the player was instructed to bring the knee forward while keeping the foot completely on the ground. The device was placed vertically on the anterior border of the tibia, just below the tibial tuberosity, determining the range of dorsiflexion in degrees. Each player had three measurements taken for each leg, obtaining an average of the three for subsequent analysis.

### 2.5. Star Excursion Balance Test (SEBT) and Y Balance Test (YBT)

Dynamic balance (SEBT) was performed (OctoBalance, Check your Motion, Albacete, Spain) to determine the dynamic stability of the lower limbs [23]. In this test, the subjects were asked to adopt a unilateral equilibrium position, executing a series of unilateral squats where they tried to reach the maximum distance possible in eight different directions with the other extremity: Three in front, two at the sides, and three at the back [24]. The current literature also includes the modified SEBT or YBT [25]. Both tests, SEBT and YBT, were performed. To be valid, each player had to: (1) Place the heel of the supporting foot on the rear edge of the octagon and the head of the second metatarsal on the front line (red), (2) keep his hands on his hips, and (3) rest only the reaching foot on the platform. The measurements were made only once per leg, since these were experienced subjects [23], and were made with both legs to determine asymmetry between both body hemispheres.

### 2.6. The 30-M Sprint Test (30S)

Linear speed was determined by performing the 30-m sprint test with the MySprint App (Apple Inc., USA) [26]. The test consists of covering a distance of 30 m at maximum speed from a standing position in which the subject is positioned behind the starting line with one foot forward. The time starts to count just as the back foot is separated from the ground with cuts at 5, 10, and 30 m and at the moment the hip passes through the marker, until the last marker is reached. Each athlete performed the test twice, with a break of at least two minutes between each attempt. Individual force–velocity relationships in sprinting were assessed with MySprint app [26]. This app uses the Samozino’s method to determine the theoretical maximum values of force (F0), velocity (V0), and power (Pmax) in sprinting [27]. Samozino’s method provides a simple method of obtaining the force–velocity relationship from the application of basic laws of motion using the running speed and the body mass of the athlete as main inputs [27]. From all the data obtained, the rate of force at 10 meters (RF-10M) and the split times of 5, 10, and 30 m of the best attempt were used for further analysis.

### 2.7. Triple Hop Test for Distance Unilateral (THTU)

The analysis of the elastic–reactive force in the horizontal vector of the lower extremities (LE) was carried out by means of the triple jump test in a unilateral manner due to the predictive power of muscle strength and the power of the LE [28,29]. Each participant positioned the leg to be measured, resting the big toe on the starting line. Once each player performed three consecutive maximum forward jumps with the same limb, the researcher measured the total distance jumped from the start to the point where the heel hit the ground upon completion of the third jump [30,31]. Arms’ swinging was allowed, and attempts were considered failed if the participant: (1) Did not complete the test as instructed, (2) lost balance during any part of the test, or (3) was unable to maintain the final posture on one limb for at least two seconds. Each athlete performed the test twice with a break of at least two minutes and the best attempt was used for further analysis.

### 2.8. Countermovement Jump (CMJ) Test 

The vertical jump test was determined using a Chronojump (Chronojump BoscoSystem^®^, Barcelona, Spain) [32]. Starting from an initial position with both feet on the ground and both hands on the hips, a squat was executed with countermovement. A jump was considered valid if it was performed with the hands on the hips, without bending the knees during flight time, and landing at the same point where the jump was made. Each player made a total of three valid attempts, and the best attempt was recorded.

### 2.9. Drop Jump Unilateral (DJU) Test 

The vertical jump test was determined using a Chronojump (Chronojump BoscoSystem^®^, Barcelona, Spain) [32]. From an initial height of 30 cm, each subject was dropped to the ground on one leg in search of the maximum application of forces reacting against the ground to jump as high as possible. Each attempt was considered valid when both hands were on the hips, the knees were not bent, and both landings were made at the same point. A total of three valid attempts were made with each leg, with a total of 30 s of recovery between each attempt. Finally, the best of these attempts was used for further analysis. The reactive strength index (RSI) was calculated using the time of flight to contact time ratio for each leg.

### 2.10. Vertical Force–Velocity Profile

The force–velocity profile was determined by the neuromuscular relationship between force and speed of the LE using CMJ with a hex bar. Each subject performed a maximum vertical CMJ jump with their body weight (BW), without loading, and then five more jumps with an extra load between 10 and 60 kg in random order. To be valid, each player had to reach at least 10 cm in height with the attempted load [33]. Before the first CMJ jump, and although the players knew the test, the execution of all jumps both without and with loading was explained to them. The CMJ without additional loading was performed as already explained in CMJ test. The differently loaded CMJs were performed with the hands holding the hex bar with the load being distributed equally between both sides of the bar, the elbows were kept extended, and the shoulders relaxed. The jump was carried out in the same way as the CMJ test. Each attempt was accompanied by two minutes of rest to ensure maximum performance.

The vertical jump test was determined using a Chronojump (Chronojump BoscoSystem^®^, Barcelona, Spain) [32]. Finally, multiple jumps were used to calculate the f–v profile, specifically the best four jumps. The data obtained were manually entered into the MyJump2 App (Apple Inc., USA) [34] to apply Samozino’s method [35] and estimate the mean force, velocity, and power of the jump considering three simple input variables: The height of the jump, body mass, and thrust distance, with the last calculated as the difference between the length of the extended leg (from the anterior superior iliac crest (ASIC) to the toes with plantar flexion) and the length from the ASIC to the ground in the squatting position. The values were F0, V0, and the f–v profile, as previously proposed [36]. Likewise, the theoretical optimal f–v profile was calculated individually for each subject, together with the imbalance in the f–v profile [37].

### 2.11. Change of Direction (COD)

The 5+5 test. Timing photocells (Chronojump BoscoSystem^®^, Barcelona, Spain) were placed to 0.5 m from starting line with a width of 2 m and 5 m from the pivot point marked on the floor, where the athlete performed changes of direction at 45°, 90°, and 180°, setting the designated leg and turning to the opposite side (side step) to go to the second time gate placed 5 m away in the shortest time possible. Each athlete had two attempts for each leg, and the best attempt was used for analysis. Between each attempt the players rested for at least two minutes.

Modified T-test. Timing photocells were placed at the starting and finish lines (Chronojump BoscoSystem^®^, Barcelona, Spain). Subjects began with both feet 0.5 m behind the starting line. Each subject was required to sprint forward as far as the first cone, located 5 m away, to the starting line and touch the base of it with the right hand. Facing forward and without crossing feet, they shuffled to the left cone and touched its base with the left hand. Subjects then shuffled to the right cone and touched its base with the right hand. Finally, they shuffled back to the left to the central cone with a lateral displacement of 2.5 m and touched its base with the left hand just before running backwards as fast as possible to the finish line [20]. The test was considered invalid if the subject crossed the feet in front of each other, did not touch the base of a cone, or did not look forward during the entire test. Each athlete had two attempts and the best attempt was used for analysis. Between each attempt the players rested for at least two minutes.

### 2.12. Statistical Analysis

Statistical analyses were performed using the SPSS version 25 statistical package for MS Windows (SPSS, Inc., Chicago, IL, USA). Data were expressed as means and standard deviations (SD). Data normality was checked using the Shapiro–Wilks test. A between-groups’ comparison analysis was carried out to determine the differences between basketball categories. Then, the COD variables were divided by the median into two groups to examine the influence of each COD upon performance: High responders (HRs), defined as players who performed above the 50 percentile for each COD, and low responders (LRs), defined as players who performed below the 50 percentile for each COD. The model was adjusted by age. The effect sizes (ES) were calculated using the Hedge’s g on the pooled SD together, and the 90% confidence intervals (CI) were calculated for all dependent variables. Probabilities were also calculated to determine whether true (unknown) differences were smaller, similar, or larger than the smallest worthwhile difference or change (0.2 × SD between subjects) [38]. Quantitative chances of better or worse effects were determined as follows: <1%, almost certainly not; 1–5%, very unlikely; 5–25%, unlikely; 25–75%, possible; 75–95%, likely; 95–99%, very likely; and >99%, most likely [39,40]. Finally, if the chances of obtaining a beneficial/better or detrimental/worse effect were both >5%, the effect was assessed as unclear [41,42]. An Excel spreadsheet downloaded from sportsci.org was used to examine comparisons between groups (xCompare2groups.xls). On the other hand, Pearson’s correlation coefficient was used to establish correlations between different CODs and sport performance variables.

## 3. Results

Table 1 shows the comparative analysis of the mean values referred to body composition, mobility, dynamic balance, linear sprint, strength, and COD according to player category. Body composition showed a greater influence of the category upon lean mass and BMI. Regarding mobility and dynamic balance, a high percentage of change was observed for the analyzed variables depending on the category. On the other hand, linear speed showed no relationship with the category except for the run time at 30 m. The strength variables, likewise, showed no relationship between them with the category except for imbalance in the f–v profile, which did show variation (27.89% [Confidence limit (CL) 90%: −10.70; 83.16]; Quantitative chances (QC) 4/25/71). Lastly, the changes observed for the COD test were greater as the angle of COD increased, with the greatest variation being recorded for COD 180° (7.05% [CL 90%: 2.33; 11.99]; QC = 0/2/98).

The comparative analysis of each COD, defined as LR and HR, and the sport performance-related variables are shown in Table 2. For linear velocity, a greater impact was observed with COD as the angle and number of CODs increased. Specifically, execution of the linear sprint in the first 10 m showed higher percentages of change for COD180° (−4.84% [CL 90%: −7.74; −1.86]) and for the T-test (−5.29% [CL 90%: −7.98; −2.52]). No significant differences were observed in the determination of the elastic–reactive force in the horizontal axis. In relation to the vertical axis elastic–reactive force for the DJU test, the group of HR players showed a greater percentage of change with respect to RSI (26.87 to 32.2% [CL 90%: 8.31; 48.61 RSI Right to 11.56; 56.66 RSI Left]) than for the height of the jump (7.5 to 17.7% [CL 90%: −3.88; 20.34 DJU R to 2.16; 35.59 DJU L]), with a greater change of this index being for the T-test, independently of the leg of execution. Finally, asymmetry analyzed in the f–v profile was determined, and a relationship was obtained with the different types of CODs evaluated. Greater changes were observed for the COD180° and T test, with the latter showing the greatest variation (−42.39% [CL 90%: −57.37; −22,16]).

**Figure 1:**
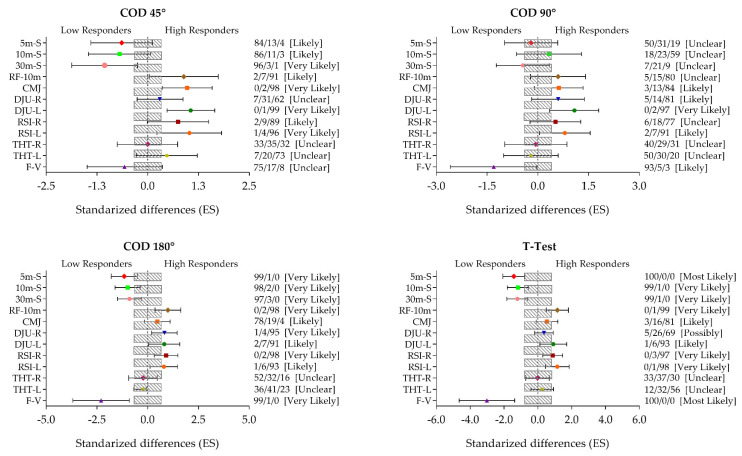
Between-group comparisons for the 5-, 10-, and 30-m linear sprint, the rate of force at 10 m, the vertical jump through countermovement jump and drop jump unilateral, the reactive strength index, the horizontal jump through triple hop test, and the vertical force–velocity profile between low responders (LH) and high responders (HR) classified by each change of direction (COD) execution time. The model was adjusted by age. Bars indicate uncertainty in the true mean changes with 90% confidence intervals. Trivial (shaded) areas were calculated from the smallest worthwhile change. Quantitative chances of better or worse effects were determined as follows: <1%, almost certainly not; 1–5%, very unlikely; 5–25%, unlikely; 25–75%, possible; 75–95%, likely; 95–99%, very likely; and >99%, most likely [33,34]. R: Right; L: Left; RF: Rate of force; CMJ: Countermovement jump; DJU: Drop jump unilateral; RSI: Reactive strength index; THT: Triple hop test; F–V: Vertical force–velocity profile; COD: Change of direction; ES: Effect size.

Practical, worthwhile differences between groups seemed evident as supported by the magnitudes of the ES and qualitative outcomes (Figure 1). The COD180° and the modified T-Test showed most likely and very likely positive effects on 5- and 10-m linear sprint (COD180°, QC 99/1/0%; T-test, QC 100/0/0%), the reactive strength index (RSI) (COD180°, 0/3/97%; T-test, QC 0/4/95%), and the vertical force–velocity profile (COD180° and T-test, QC 100/0/0%), whereas the other two CODs (COD 45° and 90°) showed unclear or likely effects.

The matrix of correlations between the different types of COD tests performed and the sports performance variables analyzed is shown in Table 3. For anthropometric measures, lean mass was positively correlated only with COD45°. With respect to linear velocity, a negative association was observed with the RF-10M variable in COD90°, COD180°, and the T-test. In contrast, a positive correlation of linear sprint speed was observed at 5 m and 10 m and the COD180° and T-test, and with the linear sprint at 30 m and the four analyzed angles. For strength, execution time at COD45° and CD180° showed a negative correlation with bilateral CMJ, DJU, and RSI. However, the execution time at COD90° and T-test were negatively correlated with DJU and RSI. Lastly, for the strength tests evaluated using the f–v profile and its analyzed variables, a positive association was observed with asymmetry, V0, and Pmax and, conversely, a negative association was found with F0 as both the number of COD or the angulation increased.

## 4. Discussion

The present study initially sought to evaluate whether the age category could influence anthropometric measurements, functional movement tests, linear sprint (30 m), and strength in basketball players. Moreover, and as the main purpose, the present study aimed to examine the relationship between the analyzed sport performance variables and the time in different COD tests and angles. Our study showed age-category differences in body composition and functional movements and planned COD test. Increases in the differences between categories were seen as the cut angle increased, and the analyzed categories were higher. On the other hand, we mainly aimed to evaluate the influence of different time-executed CODs and their angulation upon the analyzed sport performance variables. Our findings suggest an interesting relationship between performance variables and CODs. Specifically, RSI was the strength variable showing the strongest relationship with all the COD tests. Also, it is important to note that as a novelty, linear velocity and asymmetry in the f-v profile increased in relation to the angulation or number of cuts in the COD, which would confirm greater dependence upon these variables at higher angulations in the CODs.

### 4.1. Influence of Age Category on Sport Performance-Related Variables

In daily basketball practice, the physical and performance characteristics of basketball players according to age help coaches and strength-conditioning trainers to design training programs. Specifically, in basketball, anthropometric characteristics play a very important role, for example, height is considered to be the most important physical attribute and this parameter is an important factor when identifying and selecting talents [43]. This study identified differences in anthropometrics’ factors and body composition, mainly in lean mass and BMI. This idea is in accordance with previous studies that confirmed it, and also added that the greatest changes occurred between Under 14–15 and Under 17 categories [44]. Regarding positions, it must be noted that from early ages there are morphological differences between centers and guards, which serve us as references to be able to select [44], although early specialization is not recommended. In addition, other variables analyzed such as functional movements showed dependency with the age category, as several studies have claimed in young and elite basketball players [44,45], though no differences were observed in the linear sprint or strength tests, with the exception of the vertical f–v profile. In contrast with this, research [9] has shown that improved performance through vertical jumping depends on the level and category of the team sports athlete, and being more significant in basketball players especially between U14 and U17 categories [44]. Regarding the planned COD tests, increased differences between categories were seen as the cut angle increased, with better performance results in the higher categories. This could have been explained mainly by a better balance in the f–v profile of senior athletes and the relationship of this balance with changes in direction as the cut angle increased, in addition to the greater amount of lean mass in senior athletes. Similarly, for agility, it has been seen that reactive agility discriminates between basketball players of different levels [9,10], but that planned agility does not. The latter contradicts our results, but this may be due to the sample used or the type of COD test evaluated. For all aforementioned, it was clear that the age category influences anthropometric factors, but there is no clear consensus on the influence of age category on performance factors. This may be largely due to the variability of the tests used to measure performance, different performance variables assessed, or different samples used.

### 4.2. Influence of Sport Performance-Related Variables on the Time Execution COD and Angles

Basketball is an intermittent sport with demanding COD and, as each sport does, demands specific anthropometric and physical requirements. Knowledge of these requirements is of special interest to coaches and physical trainers who want to maximize the performance of their players. In this line, anthropometric variables such as body height or leg length can influence COD performance, since a shorter individual would need less time to lower the center of mass [4,7]. COD performance is based on greater acceleration of BW; therefore, a lower fat percentage together with high relative strength would benefit COD performance [46]. In this regard, the association observed between fat mass and its influence upon the type of COD45° may explain why those players with a higher fat component took longer in performing COD45°. According to our results, other studies in elite basketball players [19,47] observed the same association, but through the T-test. In addition, several studies have evidenced a weak [48] and moderate–high relationship [46,47] between BW and the T-test in athletes and elite basketball players. From a logical point of view, it seems reasonable that an athlete with a low fat percentage would perform better in COD, but what appears even more clearly is the importance of a decrease in fat mass or an increase in maximum strength without an increase in BW, which would increase the relative strength of the athlete [49].

Ankle dorsiflexion limitation observed throughout the WB-DF test suggests possible movement deterioration that may alter the mechanics of movement in multidirectional tasks [25]. Studies such as that carried out by Gonzalo-Skok et al. [25] in 15 elite male basketball players have concluded that leg asymmetry determined by the WB-DF test is related to a decrease in performance in tasks, including CODs. In contrast, the results of our study show that neither a lack of ankle mobility analyzed by the WB-DF test nor asymmetry between both legs seems to affect performance within the COD tests evaluated. Furthermore, none of the dynamic equilibrium tests—either SEBT or YBT—were found to be related to CODs, excepting left YBT with left COD90°. Other authors [25,50], however, confirmed the relationship between dynamic equilibrium and COD in a similar sport population. Although the assessment of leg asymmetry may offer a prediction of sports injuries [51], our study identified no relationship with COD performance. On analyzing each excursion separately, the only one affording high correlation with all the analyzed COD tests was found to be lower limb lateral excursion. This could be explained by the great involvement of the frontal plane in COD actions [52].

Although linear velocity and velocity in CODs are considered two different capabilities [11], the literature accepts that the former affects the latter [6]. The greater the angle of rotation, the lower the relationship with linear velocity [11]. Therefore, CODs that cover angles of less than 90° are more velocity-oriented, in contrast to angles of more than 90° that are more force-oriented [49]. In our study, angles smaller than 90° had less effect on the variation in linear velocity and, contrary to scientific evidence, we observed that the relationship between linear velocity and COD increased at the same time as the COD angle or the number of cuts. These contradictions could be solved by introducing the concept of deficit in COD, where COD action is isolated from linear action within the tests [53]. While several studies [54,55] have shown the relationship of CODs to the deficit itself, no considerations have been made with linear velocity. Although some authors [56] still suggest linear velocity to be a determining factor for COD performance, high COD approaching velocities may increase the loads which the knee receives in valgus. So, it should be important to seek optimal balance between force and speed in order to minimize the drop in velocity prior to COD [56]. Likewise, in relation to linear velocity, the application of forces in the horizontal vector during sprint determined by the variable RF-10M showed high correlation with COD90°. In line with this, other studies [14,57] have found that those team sports’ athletes who performed better in the COD 505 test showed higher maximum horizontal propulsion forces and shorter ground contact times, as well as equally higher approaching velocities and greater speed reductions during cutting action. The key to better performance in COD actions is to minimize contact times in the field [14], and strength affords the ability to do so. High horizontal propulsion forces and low vertical impact forces in the last step, together with high braking forces in the penultimate step, are associated to higher COD performance [14]. Faster female basketball athletes produce higher braking forces, helping to increase the starting speed during COD due to the storage and utilization of elastic energy, and propulsion [19]. Therefore, it is clear that strength is a determining factor for improving performance in COD. However, the scientific literature does not clarify which of the different manifestations is the most determining, since COD is a dynamic and complex action that involves application of the stretching–shortening cycle in a vertical and horizontal axis unilaterally.

In relation to vertical force through the CMJ, DJU, and CODs, the literature affords contradictory results with CMJ [16], mainly due to gender differences [46]. Our results would partially confirm this relationship of CMJ with COD45° and COD180°. Regarding the relationship between COD and DJU, we observed that both variables have similar technical execution mechanisms and were significantly related, as also confirmed by Young et al. [11]. Interestingly, however, in our study we found RSI to be more determinant for COD than the height of the jump, since this index is determined by capacity in the application of forces with low contact times [14]. In this respect, a maximum force development of approximately 0.44–0.72 s is required for COD [58]. When studying the association with force in the horizontal axis by THTU, we found no statistically significant associations with the different CODs evaluated. In addition to COD performance with bilateral vertical and unilateral horizontal jumping, recent research [59] has determined a similar muscle activation pattern during both actions, though activation of the long adductor, biceps femoris, and semitendinosus muscles was increased in COD actions to stabilize the hip and slow knee joint movements when turning compared to jumping. On the other hand, asymmetry assessed in all the tests performed in single mode revealed no negative impact on the performance of basketball players (data not shown), as confirmed by other studies, and points to the need to clarify the magnitude of changes in COD performance that can be explained by asymmetries [60].

During COD, the use of the stretching–shortening cycle requires all phases of muscle contraction (concentric, eccentric, and isometric) to be present and to develop in similar proportions [19]. For this reason, we considered that it would not be appropriate to evaluate the different manifestations of force in an isolated manner. We, therefore, hypothesized that the influence of the force and speed imbalance with the f–v profile could have an impact on COD capacity. To our knowledge, the present study is the first to evidence this relationship. Interestingly, this profile creates an individualized graphic representation of the force that can be applied to different loads, it being possible to obtain an optimal profile in which maximum power against BW may be achieved during a vertical jump [36]. When working on reducing this imbalance between force and speed, the neuromuscular system is optimized, securing maximum power in ballistic movements with BW itself, such as jumping or sprinting [37]. So, it seems logical that improvements are also obtained in terms of COD performance. Our hypothesis would be confirmed by the results showing a strong correlation between the different variables obtained from the f–v profile and the COD test, with this relationship being stronger the greater the angle of change and/or the number of cuts. Therefore, not only are improvements in the application of forces with BW itself achieved in the face of changes with sharper angles, which will be the most demanding of forces [56], but also the fatigue generated by several consecutive BWs is reduced - as occurs in real game action - and performance is improved.

Finally, COD and agility movements are multidimensional skills involving numerous variables to produce a faster performance [4] and its performance depends on the specific sport and position [10], making it challenging to asses these variables all at once. Despite the usefulness of these findings, the present study has some limitations that should be addressed. Firstly, the authors focused on analyzing the variables that contribute to the performance of the COD, to a greater or lesser extent, exposed throughout the study, but the performance of the COD is multifactorial, as abovementioned. So, some factors such as electromyographic muscle activation were not investigated and are limitations of this study. In addition, this research was inherently limited by its cross-sectional design and the size of sample. Although these findings are limited to a small sample, it was performed with basketball players from two different age categories who trained in the same basketball club, following the same physical, technical, and tactical training programs. This certainly reinforces the applicability and relevance of our findings.

## 5. Conclusions

To summarize, the present study evidences age-category differences in body composition, functional movements, and planned COD test. Moreover, those differences were larger with increasing the cutting angle and the age category. In contrast, although body composition, mobility, and balance were not relevant to COD, both linear velocity and strength showed a strong association with COD at sharper angles. Specifically, the unilateral strength test in the vertical vector seems best option for determining COD performance, probably due to the reactive strength and the time of contact with the field, required for optimal force application. Lastly, the large changes determined by the f–v profile for CODs afford new insight to the enhancement of COD performance. Therefore, this study shows the relevance of the f–v profile in order to design training programs to optimize not only jumping and sprinting performance, as already studied, but also performance in COD actions, although more research is needed to confirm this.

## 6. Practical Application

Coaches must know the specific movement demands of each sport, and, as has been previously demonstrated, basketball is a sport in which fast actions such as jumping and COD are particularly important, with performance in these types of actions making the difference in the final result. The findings of the present study demonstrate the relationship between the f–v profile and the improvement of results in those actions that are determinant for performance of the sport. Therefore, coaches must carry out intervention programs with their players, with the aim of improving the optimal and individualized f–v profile of each player. This will allow better development of power for each athlete, resulting in improved performance of each individual.

## Figures and Tables

**Table 1:** Data description referred to body composition, mobility, dynamic balance, linear sprint, strength, and change of direction according to player category.

Variables	JUNIOR		SENIOR		% (CL 90%)	ES (CL 90%)	Chances (%)	Outcome
	Mean	SD	Mean	SD				
**Body Composition**								
BMI (kg/cm^2^)	21.8	2.90	24.1	2.87	−9.65 (−17.81; −0.68)	−0.81 (−1.57; −0.05)	91/7/2	Likely
Lean mass (kg)	35.9	4.12	42.8	4.45	15.83 (−22.44; −8.67)	−1.58 (−2.33; −0.83)	100/0/0	Most likely
Fat mass (%)	11.9	6.61	11.4	5.08	−5.77 (−36.02; 38.77)	−0.13 (−0.95; 0.70)	44/31/25	Unclear
** Mobility **								
WB-DF R (°)	40.7	5.65	37.1	4.31	9.86 (0.19; 20.45)	0.78 (0.02; 1.54)	2/8/90	Likely
WB-DF L (°)	40.1	5.60	38.4	3.77	3.77 (−5.68; 14.15)	0.34 (−5.54; 1.22)	15/24/61	Unclear
** Dynamic Balance **								
SEBT R (cm)	57.6	7.31	63.5	4.97	−8.77 (−15.67; −1.30)	−1.08 (−2.00; −0.15)	94/4/1	Likely
SEBT L (cm)	57.3	7.60	63.2	6.27	−8.80 (−16.62; −0.25)	−0.85 (−1.67; −0.02)	90/7/2	Likely
YBT R (cm)	62.9	7.05	69.0	6.34	−7.94 (−14.69; −0.66)	−0.81 (−1.56; −0.06)	91/7/2	Likely
YBT L (cm)	62.8	7.97	67.9	7.79	−6.73 (−14.93; 2.26)	−0.56 (−1.29; 0.18)	79/16/5	Likely
** Linear Sprint **								
5 m sprint (s)	0.89	0.04	0.88	0.04	0.87 (−2.46; 4.31)	0.17 (−0.49; 0.84)	17/36/47	Unclear
10 m sprint (s)	1.48	0.06	1.47	0.08	0.87 (−2.69; 4.57)	0.15 (−0.48; 0.78)	17/38/45	Unclear
30 m sprint (s)	4.31	0.14	4.16	0.19	3.18 (−0.23; 6.71)	0.58 (−0.04; 1.20)	2/13/85	Likely
RF-10M	0.30	0.01	0.31	0.02	−2.33 (−5.87; 1.33)	−0.41 (−1.04; 0.23)	71/23/6	Unclear
** Strength **								
CMJ (cm)	34.9	5.14	35.6	4.78	−0.51 (−10.45; 10.54)	−0.03 (−0.72; 0.65)	34/38/28	Unclear
DJU R (cm)	17.5	3.41	17.6	1.00	−1.93 (−12.55; 9.97)	−0.32 (−2.21; 1.57)	55/14/32	Unclear
DJU L (cm)	17.1	3.45	16.3	3.40	5.63 (−10.06; 24.05)	0.23 (−0.45; 0.91)	14/33/53	Unclear
RSI R	0.93	0.28	0.99	0.21	7.78 (−25.03; 13.42)	−0.32 (−1.13; 0.50)	60/26/14	Unclear
RSI L	0.93	0.31	0.87	0.25	4.79 (−16.89; 32.14)	0.15 (−0.59; 0.89)	21/33/45	Unclear
THT R (cm)	566.3	42.4	566.1	59.5	1.64 (−4.90; 8.62)	0.14 (−0.43; 0.70)	16/42/42	Unclear
THT L (cm)	570.3	58.2	598.5	63.9	−2.96 (−9.70; 4.29)	−0.26 (−0.87; 0.36)	56/33/11	Unclear
F-V	61.8	17.3	51.0	20.5	27.89 (−10.70; 83.16)	0.37 (−0.17; 0.91)	4/25/71	Possibly
** COD **								
COD45° R (s)	2.01	0.09	2.03	0.11	−1.81 (−4.96; 1.44)	−0.33 (−0.91; 0.26)	65/29/7	Unclear
COD45° L (s)	2.02	0.08	2.05	0.16	−1.52 (−5.94; 3.09)	−0.19 (−0.74; 0.37)	48/40/12	Unclear
COD90° R (s)	2.37	0.13	2.26	0.12	4.40 (0.24; 8.73)	0.77 (0.04; 1.49)	2/8/90	Likely
COD90° L (s)	2.35	0.09	2.27	0.10	3.00 (−0.14; 6.23)	0.63 (−0.03; 1.28)	2/12/86	Likely
COD180° R (s)	2.75	0.16	2.61	0.10	4.68 (0.90; 8.59)	1.12 (0.22; 2.02)	1/4//95	Very likely
COD180° L (s)	2.77	0.18	2.58	0.14	7.05 (2.33; 11.99)	1.19 (0.40; 1.97)	0/2/98	Very likely
COD T-test (s)	6.75	0.39	6.49	0.34	3.48 (−0.67; 7.80)	0.61 (−0.12; 1.35)	4/14/83	Likely

BMI: Body mass index; R: Right; L: Left; WB-DF: Weight-bearing dorsiflexion; SEBT: Star excursion balance test; YBT: Y balance test; RF: Rate of force; CMJ: Countermovement jump; DJU: Drop jump unilateral; RSI: Reactive strength index; THT: Triple hop test; f-v: Vertical force–velocity profile; COD: Change of direction; SD: Standard deviation; CL: Confidence limits; ES: Effect size.

**Table 2:** Comparison of the 5, 10, and 30 m linear sprint, the rate of force at 10 m, the vertical jump through countermovement jump and drop jump unilateral, the reactive strength index, the horizontal jump through triple hop test, and the vertical force–velocity profile between low responders (LH) and high responders (HR) classified by each change of direction (COD) execution time.

Variables	LR		HR		% (CL 90%)	ES (CL 90%)
	Mean	SD	Mean	SD		
**COD45°**						
5 m sprint (s)	0.90	0.04	0.87	0.04	−2.43 (−5.23; 0.46)	−0.64 (−1.40; 0.12)
10 m sprint (s)	1.50	0.07	1.45	0.07	−2.66 (−5.52; 0.29)	−0.69 (−1.46; 0.07)
30 m sprint (s)	4.15	0.17	4.33	0.13	−3.86 (−6.73; −0.91)	−1.06 (−1.87; −0.25)
RF-10M	0.30	0.01	0.31	0.02	3.53 (0.16; 7.02)	0.89 (0.04; 1.74)
CMJ (cm)	32.6	4.41	37.7	4.00	15.97 (5.57; 27.39)	0.97 (0.36; 1.59)
DJU R (cm)	17.1	2.97	17.9	2.04	6.21 (−5.10; 18.87)	0.30 (−0.26; 0.87)
DJU L (cm)	14.6	2.77	18.6	2.74	26.81 (11.22; 44.59)	1.06 (0.48; 1.65)
RSI R	0.86	0.19	1.04	0.27	20.04 (−0.30; 44.52)	0.75 (−0.01; 1.50)
RSI L	0.77	0.17	1.03	0.30	29.20 (5.92; 57.58)	1.03 (0.23; 1.82)
THT R (cm)	564.9	45.8	567.4	55.7	−0.02 (−6.76; 7.21)	0.00 (−0.75; 0.74)
THT L (cm)	569.2	50.7	597.1	69.0	5.03 (−2.87; 13.57)	0.47 (−0.28; 1.22)
F-V	61.5	18.0	52.2	20.0	−19.95 (−44.29; 15.05)	−0.57 (−1.49; 0.36)
**COD90°**						
5 m sprint (s)	0.88	0.04	0.88	0.04	−0.93 (−4.56; 2.84)	−0.20 (−0.98; 0.59)
10 m sprint (s)	1.47	0.06	1.48	0.08	1.35 (–2.54; 5.40)	0.33 (−0.63; 1.29)
30 m sprint (s)	4.29	0.16	4.19	0.18	−2.19 (−5.96; 1.73)	−0.44 (−1.23; 0.34)
RF-10M	0.30	0.01	0.31	0.02	3.06 (−1.09; 7.38)	0.60 (−0.22; 1.41)
CMJ (cm)	33.7	5.51	36.6	3.94	12.25 (−1.93; 28.48)	0.62 (−0.10; 1.34)
DJU R (cm)	16.5	2.22	18.5	2.41	9.83 (−2.72; 24.01)	0.60 (−0.18; 1.38)
DJU L (cm)	15.3	3.40	18.0	2.91	30.40 (8.97; 56.05)	1.08 (0.35; 1.80)
RSI R	0.84	0.25	1.06	0.20	17.03 (−6.83; 47.00)	0.52 (−0.23; 1.27)
RSI L	0.81	0.29	0.99	0.25	34.59 (2.05; 77.50)	0.80 (0.05; 1.55)
THT R (cm)	563.1	43.7	569.1	57.1	−0.53 (−8.05; 7.62)	−0.06 (−0.98; 0.86)
THT L (cm)	581.2	64.3	586.1	61.1	−2.41 (−11.37; 7.45)	−0.20 (−1.01; 0.60)
F-V	64.9	15.7	49.0	19.5	−30.84 (−51.75; −0.88)	−1.30 (−2.58; −0.03)
**COD180°**						
5 m sprint (s)	0.90	0.04	0.86	0.03	−4.39 (−6.74; −1.99)	−1.15 (−1.79; −0.51)
10 m sprint (s)	1.51	0.07	1.44	0.05	−4.84 (−7.74; −1.86)	−0.99 (−1.60; −0.37)
30 m sprint (s)	4.35	0.19	4.13	0.10	−4.47 (−7.31; −1.54)	−0.89 (−1.48; −0.30)
RF-10M	0.30	0.01	0.31	0.01	5.05 (1.83; 8.38)	1.00 (0.37; 1.64)
CMJ (cm)	33.82	5.11	36.54	4.46	8.36 (−2.46; 20.37)	0.48 (−0.15; 1.11)
DJU R (cm)	16.39	2.18	18.60	2.39	13.69 (3.23; 25.22)	0.83 (0.20; 1.45)
DJU L (cm)	15.32	2.65	17.99	3.55	16.61 (0.97; 34.68)	0.82 (0.05; 1.58)
RSI R	0.84	0.23	1.06	0.22	27.61 (9.17; 49.18)	0.91 (0.33; 1.49)
RSI L	0.79	0.20	1.00	0.31	25.44 (3.46; 52.09)	0.80 (0.12; 1.47)
THT R (cm)	572.4	47.8	560.6	53.5	−2.14 (−8.61; 4.78)	−0.22 (−0.93; 0.48)
THT L (cm)	586.9	66.4	580.8	59.0	−0.91 (−8.75; 7.60)	−0.07 (−0.68; 0.55)
F-V	69.2	12.7	45.1	17.0	−40.19 (−56.19; −18.33)	−2.29 (−3.68; −0.90)
**T-Test**						
5 m sprint (s)	0.91	0.03	0.86	0.03	−4.88 (−7.02; −2.68)	−1.42 (−2.06; −0.77)
10 m sprint (s)	1.52	0.06	1.44	0.05	−5.29 (−7.98; −2.52)	−1.18 (−1.80; −0.55)
30 m sprint (s)	4.37	0.16	4.11	0.11	−5.29 (−7.88; −2.63)	−1.21 (−1.83; −0.60)
RF-10M	0.30	0.01	0.31	0.01	5.46 (2.34; 8.68)	1.16 (0.50; 1.81)
CMJ (cm)	33.7	4.96	36.7	4.52	9.15 (−1.65; 21.14)	0.54 (−0.10; 1.18)
DJU R (cm)	17.0	3.07	18.1	1.84	7.55 (−3.88; 20.34)	0.36 (−0.19; 0.91)
DJU L (cm)	15.2	2.49	18.1	3.58	17.69 (2.16; 35.59)	0.91 (0.12; 1.70)
RSI R	0.84	0.23	1.06	0.22	26.87 (8.31; 48.61)	0.88 (0.29; 1.46)
RSI L	0.76	0.17	1.03	0.30	32.20 (11.56; 56.66)	1.15 (0.45; 1.85)
THT R (cm)	566.5	47.7	566.0	54.3	−0.17 (−6.67; 6.79)	−0.02 (−0.72; 0.68)
THT L (cm)	573.9	60.3	592.8	63.4	3.28 (−4.75; 11.99)	0.26 (−0.40; 0.92)
F-V	70.2	11.0	44.2	16.6	−42.39 (−57.37; −22,16)	−3.01 (−4.65; −1.37)

The model was adjusted by age. R: Right; L: Left; RF: Rate of force; CMJ: Countermovement jump; DJU: Drop jump unilateral; RSI: Reactive strength index; THT: Triple hop test; F–V: Vertical force–velocity profile; COD: Change of direction; SD: Standard deviation; CL: Confidence limits; ES: Effect size; LR: Low responder; HR: High responder.

**Table 3:** Association matrix between COD and body composition, mobility, dynamic balance, linear sprint, and strength.

Variables	COD45°		COD90°		COD180°		COD
	L (s)	R (s)	L (s)	R (s)	L (s)	R (s)	T-Test (s)
**Body Composition**							
BMI (kg/height)	0.165	0.278	−0.075	−0.052	−0.048	−0.203	−0.226
**Lean mass (kg)**	0.167	−0.076	−0.341	−0.300	−0.380	−0.374	−0.370
Fat mass (%)	0.193	0.527 **	0.246	0.154	0.384	0.184	0.100
**Mobility**							
WB-DF R (°)	−0.056	−0.128	0.089	−0.008	−0.011	0.058	0.055
WB-DF L (°)	−0.121	0.144	0.316	0.196	0.085	0.132	0.128
**Dynamic Balance**							
SEBT L (cm)	0.222	−0.187	−0.394	−0.086	−0.237	−0.193	−0.171
SEBT R (cm)	0.132	−0.226	−0.403	−0.187	−0.334	−0.262	−0.148
YBT L (cm)	0.211	−0.144	−0.414 *	−0.139	−0.231	−0.153	−0.141
YBT R (cm)	0.099	−0.240	−0.430 *	−0.251	−0.383	−0.269	−0.178
**Linear Sprint**							
5 m sprint (s)	0.176	0.125	0.227	−0.050	0.382	0.476 *	0.456 *
10 m sprint (s)	0.153	0.104	0.233	−0.037	0.404	0.461 *	0.427 *
30 m sprint (s)	0.164	0.355 *	0.441 *	0.214	0.498 *	0.537 **	0.501 *
RF-10M	−0.180	−0.339	−0.433 *	−0.169	−0.470 *	−0.514 *	−0.472 *
**Strength**							
CMJ (cm)	−0.215	−0.668 **	−0.340	−0.226	−0.462 *	−0.443 *	−0.314
DJU L (cm)	−0.293	−0.632 **	−0.532 **	−0.273	−0.451 *	−0.400	−0.541 **
DJU R (cm)	−0.261	−0.162	−0.294	−0.170	−0.451 *	−0.515 *	−0.360
RSI L	−0.368	−0.446 *	−0.427 *	−0.246	−0.504 *	−0.420 *	−0.626 **
RSI R	−0.265	−0.378	−0.465 *	−0.339	−0.546 **	−0.579 **	−0.656 **
THT R (cm)	0.232	−0.295	−0.289	0.057	0.107	0.053	−0.129
THT L (cm)	−0.284	−0.196	−0.311	−0.245	−0.034	−0.036	−0.121
**F-V Profile**							
F-V Imbalance	0.183	0.255	0.603 **	0.407	0.523 *	0.589 **	0.784 **
F-V V0	0.219	0.171	0.379	0.330	0.441 *	0.530 **	0.680 **
F-V F0	−0.344	−0.330	−0.711 **	−0.555 **	−0.605 **	−0.621 **	−0.778 **
F-V Pmax	0.097	0.065	0.193	0.166	0.244	0.342	0.468 *

BMI: Body mass index; R: Right; L: Left; WB-DF: Weight-bearing dorsiflexion; SEBT: Star excursion balance test; YBT: Y balance test; RF: Rate of force; CMJ: Countermovement jump; DJU: Drop jump unilateral; RSI: Reactive strength index; THT: Triple hop test; F–V: Vertical force–velocity profile; V0: theoretical maximal unloaded velocity; F0: Theoretical maximal force; Pmax: Maximal power; COD: Change of direction; * *p* < 0.05; ** *p* < 0.001.
